# A recurrent single-amino acid deletion (p.Glu500del) in the head domain of ß-cardiac myosin in two unrelated boys presenting with polyhydramnios, congenital axial stiffness and skeletal myopathy

**DOI:** 10.1186/s13023-022-02421-7

**Published:** 2022-07-19

**Authors:** Ingrid Bader, M. Freilinger, F. Landauer, S. Waldmüller, W. Mueller-Felber, C. Rauscher, W. Sperl, R. E. Bittner, W. M. Schmidt, J. A. Mayr

**Affiliations:** 1 Clinical Genetics Unit, University Hospital, Salzburger Landeskliniken and Paracelsus Medical University Salzburg, 5020 Salzburg, Austria; 2 University Children’s Hospital, Salzburger Landeskliniken and Paracelsus Medical University Salzburg, 5020 Salzburg, Austria; 3 University Clinic of Orthopaedic and Trauma Surgery, SALK and Paracelsus Medical University Salzburg, 5020 Salzburg, Austria; 4 Neuromuscular Research Department, Center of Anatomy and Cell Biology, Medical University of Vienna, 1090 Vienna, Austria; 5 Universitätsklinik Für Kinder- Und Jugendheilkunde, Medical University of Vienna, 1090 Vienna, Austria; 6 Dr. V. Hauner Children’s Hospital, Ludwig-Maximilian University of Munich, Munich, Germany; 7 Institute of Medical Genetics and Applied Genomics, University of Tübingen, Calwerstr. 7, 72076 Tübingen, Germany

**Keywords:** *MYH7*, Myopathy, ß-cardiac myosin heavy chain, Actin-binding domain, Exome-analysis

## Abstract

**Background:**

Alterations in the *MYH7* gene can cause cardiac and skeletal myopathies. *MYH7*-related skeletal myopathies are extremely rare, and the vast majority of causal variants in the *MYH7* gene are predicted to alter the rod domain of the of ß-cardiac myosin molecule, resulting in distal muscle weakness as the predominant manifestation. Here we describe two unrelated patients harboring an in-frame deletion in the *MYH7* gene that is predicted to result in deletion of a single amino acid (p.Glu500del) in the head domain of ß-cardiac myosin. Both patients display an unusual skeletal myopathy phenotype with congenital axial stiffness and muscular hypertonus, but no cardiac involvement.

**Results:**

Clinical data, MRI results and histopathological data were collected retrospectively in two unrelated boys (9 and 3.5 years old). Exome sequencing uncovered the same 3-bp in-frame deletion in exon 15 (c.1498_1500delGAG) of the *MYH7* gene of both patients, a mutation which deletes a highly conserved glutamate residue (p.Glu500del) in the relay loop of the head domain of the ß-cardiac myosin heavy chain. The mutation occurred de novo in one patient, whereas mosaicism was detected in blood of the father of the second patient. Both boys presented with an unusual phenotype of prenatal polyhydramnios, congenital axial stiffness and muscular hypertonus. In one patient the phenotype evolved into an axial/proximal skeletal myopathy without distal involvement or cardiomyopathy, whereas the other patient exhibited predominantly stiffness and respiratory involvement. We review and compare all patients described in the literature who possess a variant predicted to alter the p.Glu500 residue in the ß-cardiac myosin head domain, and we provide in-silico analyses of potential effects on polypeptide function.

**Conclusion:**

The data presented here expand the phenotypic spectrum of mutations in the *MYH7* gene and have implications for future diagnostics and therapeutic approaches.

**Supplementary Information:**

The online version contains supplementary material available at 10.1186/s13023-022-02421-7.

## Background

The *MYH7* gene is located on the long arm of chromosome 14 and encodes the heavy chain of ß-cardiac myosin, which is expressed in both cardiac and slow skeletal muscle fibers. Mutations in the *MYH7* gene cause a spectrum of clinically heterogeneous cardiac myopathies, including hypertrophic cardiomyopathy (HCM), dilated cardiomyopathy and left ventricular noncompaction, as well as an array of skeletal myopathies, including Laing distal myopathy, myosin storage myopathy and scapuloperoneal myopathy (Additional file 1: Table S1).

*MYH7* missense mutations located in the head domain of ß-cardiac myosin are a frequent cause of HCM [1]. In contrast, skeletal myopathies caused by *MYH7* mutations are rare, and the causal mutations are predominantly located in the rod domain [2–4].

Three studies of larger cohorts of patients with *MYH7*-related skeletal myopathies suggest a continuum of clinical manifestations [5–7].

The study by Lamont et al. examined 88 patients from 21 families [7]. In all families there was at least one affected member who had onset of symptoms in childhood. Two children presented as floppy babies and two children had congenital cardiomyopathy. Cardiomyopathy, in addition to skeletal myopathy, was present in members from 9 of the 21 families. Distal weakness (foot drop) was the most frequent manifestation, present in 81% of all individuals. Spinal involvement (scoliosis or rigidity) was present in 57% of the cases. All mutations reported by Lamont et al. were located in the rod domain [7].

The study by Fiorillo et al. investigated 21 cases from 15 families [6]. In nine of the 15 patients, onset of symptoms (predominantly distal weakness) occurred in childhood. Cardiomyopathy, in addition to the skeletal myopathy, was present in members from 7 of the 15 families. Distal weakness (foot drop) was the most frequent manifestation, present in 18 of 21 individuals. Spinal involvement (scoliosis or rigidity) was present in 11 of 21 individuals. All but two of the mutations were located in the rod domain; the two exceptions (p.Thr441Met and p.Leu594Met) were both located in the head domain, and the two affected individuals exhibited distal weakness [6].

The study by Dabaj et al. focused on eight patients, 12 to 43 years of age, all of whom showed severe axial involvement. Earliest age of onset was 18 months, evidenced by waddling gait and frequent falls. Distal weakness was present in all eight patients and cardiomyopathy was absent in all eight. Scoliosis, rigidity and stiffness were reported in six patients. All mutations detected by Dabaj et al. were missense mutations located in the rod domain [5].

Here, we show MRI results and describe clinical and histopathological data of two unrelated boys, both of whom presented prenatally with polyhydramnios and both of whom were symptomatic postnatally, exhibiting muscular hypertonia and congenital axial stiffness, but not muscular hypotonia or floppiness as previously described in other cases [8].

Exome analyses of DNA isolated from peripheral blood of both patients revealed a heterozygous 3-bp in-frame deletion in *MYH7*, predicted to result in a single amino acid deletion (p.Glu500del) in the myosin head domain. The deletion occurred de novo in one patient and was detected as mosaicism in blood of the father of the other patient.

## Materials and methods

### Patients

We retrospectively collected clinical, histopathological and MRI data of two unrelated patients with muscular disorders and an identical heterozygous in-frame 3-bp deletion in exon 15 of *MYH7* (NM_000257.4): c.[1498_1500delGAG];[ =]; (p.[Glu500del];[ =]) detected by exome sequencing. Both patients are of Austrian descent. Both were seen as newborns at the Children’ s University Hospital of the Medical University of Vienna and one patient was also seen at 9 years of age at the University Clinic of Orthopedic and Trauma Surgery in Salzburg. The parents provided written informed consent.

### Exome sequencing, sequence analysis and segregation analysis

Exome analysis from blood of patient 1 was performed as reported previously [9]. Briefly, DNA was isolated from peripheral blood. The library was prepared by SureSelect60Mbv6 (Agilent) and paired-end sequenced on a HiSeq 4000 platform (Illumina) with a read length of 100 bases. The sequence reads were aligned to the human genome assembly hg19 by using the Burrows-Wheeler Aligner (BWA, v.0.5.87.5). Detection of genetic variation was performed using SAMtools (v 0.1.18), PINDEL (v 0.2.4t), and ExomeDepth (v 1.0.0). The cut-off for biallelic inheritance was 1% minor allele frequency; for dominant inheritance, only variants with a minor allele frequency of 0.1% were further considered. For comparison, data of 25,183 exomes were available in the in-house database of the Munich exome server, which includes patients with different medical conditions and also clinically unaffected parents. Variants below the cut-off were further filtered for genes annotated in the OMIM database that matched the term “myopathy”. After this filter-step, 12 genes remained (*KAT6B, MYBPC1, MAN2B1, CLCN6, MYO18B, DYSF, DCTN1, TTN, OPA1, DYSF, AR* and *MYH7*). Based on clinical information, inheritance pattern and frequency in unaffected individuals, only *MYH7* remained as a candidate for follow-up.

Exome analysis in patient 2 was performed from blood. DNA libraries were prepared using SureSelectXT Human All Exon V6 (Agilent Technologies) and sequenced on an Illumina HiSeq 2500 sequencing system with 125-bp paired-end reads. All library preparation and sequencing steps were performed at GATC Biotech AG (Constance, Germany). Sequences were analyzed at the Neuromuscular Research Department using an in-house developed bioinformatics pipeline, starting from FASTQ files. Reads were mapped to the human genome reference sequence (build hg19) using the BWA-MEM algorithm. Within the targeted coding exons, the average depth of coverage was 60-fold, with 91.8% of target sequence covered at least tenfold. Variant calling was performed using the Genome Analysis Toolkit (GATK) Haplotype Caller. Variant annotation and comparison to reference databases (1000 genomes project, NHLBI GO exome Sequencing Project, ExAC 0.3, gnomAD Browser 2.0, and an in-house database comprising > 500 individuals) were conducted using ANNOVAR and exported to Excel spreadsheets for final analysis and variant prioritization. Filtering for potentially damaging variants in genes known to be causatively involved in neuromuscular disorders and fulfilling a minor allele frequency cut-off of < 0.1% in reference datasets resulted in 540 variants, of which 14 were regarded as potentially pathogenic and affected genes known to be causatively involved in neuromuscular diseases; of these, 4 genes were known to be associated with myopathy (*FLNC*, *POMK*, *KCNE1*, and *MYH7*).

Based on clinical information and mode of inheritance, the variant in *MYH7* was the only candidate that remained for follow-up.

In peripheral blood of both unrelated patients, a heterozygous in-frame 3-bp deletion in exon 15 of *MYH7* (NM_000257.4): c.[1498_1500delGAG];[ =]; (p.[Glu500del];[ =]) was detected. The variant is deposited in the ClinVar-Database under the accession ClinVar; [VCV000525034.2]. Segregation analysis in blood of the parents was performed by targeted standard Sanger sequencing. Multiple Sequence Alignment (MSA) was performed using Clustal Omega, PSI-Blast and the Conserved Domain Database (CDD) [10]. Structural in silico prediction of the effect of the variant was performed using the program AlphaFold[11].

### Muscle biopsy

Both patients underwent a muscle biopsy within their first two years of life. Specimens were stained by routine histological and histochemical methods and by standard immunohistochemical techniques [12].

## Results

### Patients and clinical features

**Fig. 1:**
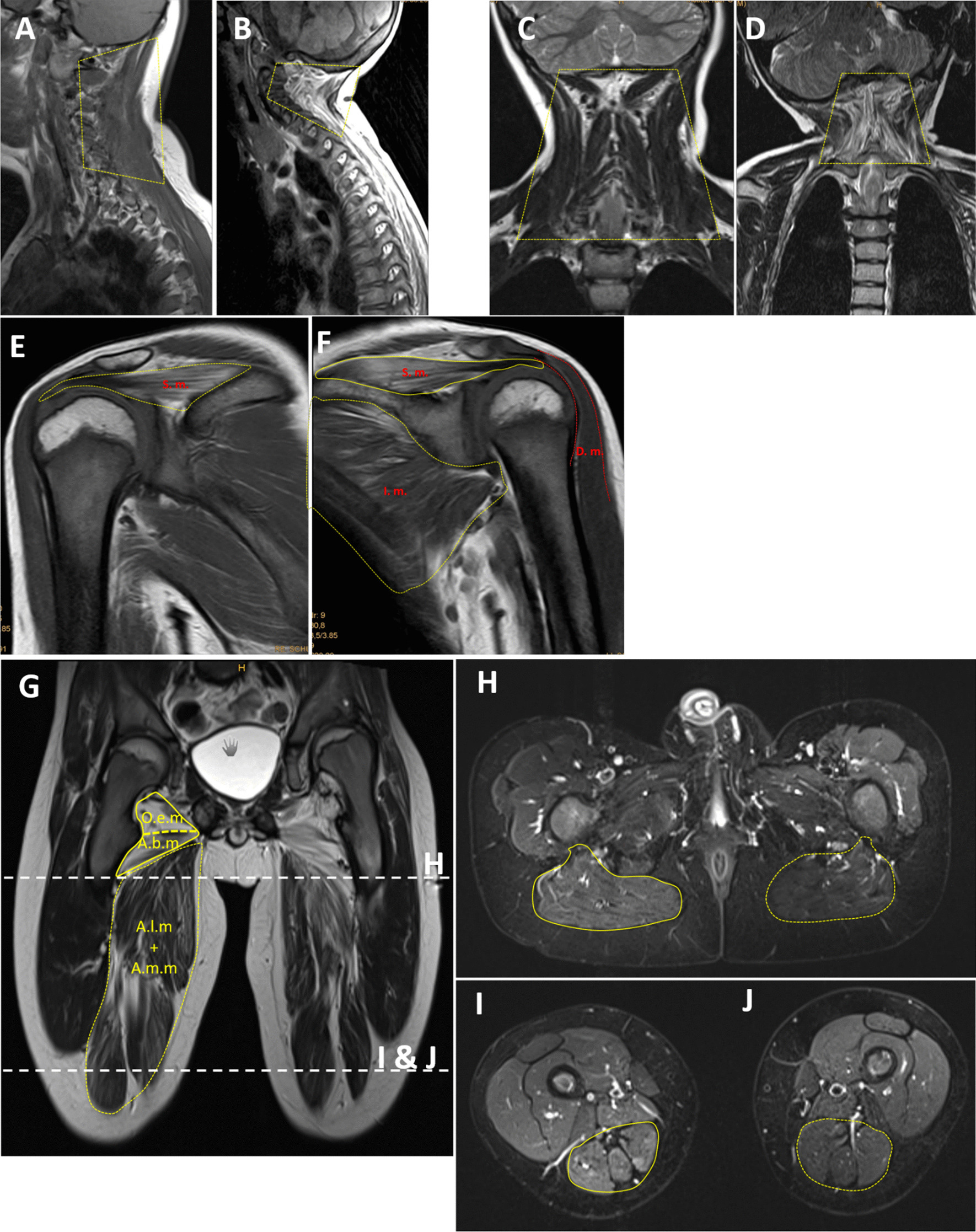
MRI findings of patient 1. Top row, T2 w MRI. (B + D) Sagittal (**B**) and frontal (**D**) view of the neck muscles of patient 1 at the age of 7 years. Neck muscles and paravertebral muscles are degenerated with compensatory hyperlordosis of the cervical spine compared to (A + C) age-matched healthy controls. Second row shows the difference between right and left T2 w MRI at the age of 7 years: (E + F) frontal view of right + left shoulder showing symmetrical fatty degeneration of the supraspinatus muscle (S.m.) and involvement of the infraspinatus muscles (I.m.) compared to normal signal for deltoid muscles (D.m. in red). Bottom row shows the difference between right and left T1 w MRI at the age of 9 years: (**G**) Frontal view of thigh with obturator internus (O.a.m.) and adductor brevis (A.b.m) muscles replaced by fat tissue, adductor longus (A.l.m.) and adductor magnus (A.m.m.) muscles fatty degenerated. (**H**) A proximal axial view of the hip muscles: with the left gluteus maximus muscle (G.m.m.) showing slight signs of fatty infiltration, the right side appears not to be affected. (I + J) A distal axial view of the thigh muscle showing very slight involvement of the left biceps femoris, sartorius and semimembranosus muscles (circled in yellow) and no obvious involvement of the right thigh

Patient 1 (Table 1) is a nine-year-old boy and the first child born to healthy, non-consanguineous parents of Austrian descent. Family history was unremarkable, gestation was complicated by excess amniotic fluid and gestational diabetes. Patient 1 was born at term by elective Caesarean section. Anthropometric parameters were normal. Hip dysplasia was present at birth and treated by surgery. Within the first month of life, muscular hypertonia, stiffness and reduced movements in the shoulder-girdle were evident. Electrophysiological investigations gave normal results. Startle-disease (stiff-baby syndrome) was considered as a differential diagnosis and a therapy trial with clonazepam was initiated. An MRI of the cervical spine and the shoulder joints at age 7 years revealed generalized atrophy of the neck muscles and the paravertebral cervical muscles as well as atrophy of distinct muscles of the shoulder girdle (Fig. 1B, D, E, F).

**Table 1:**
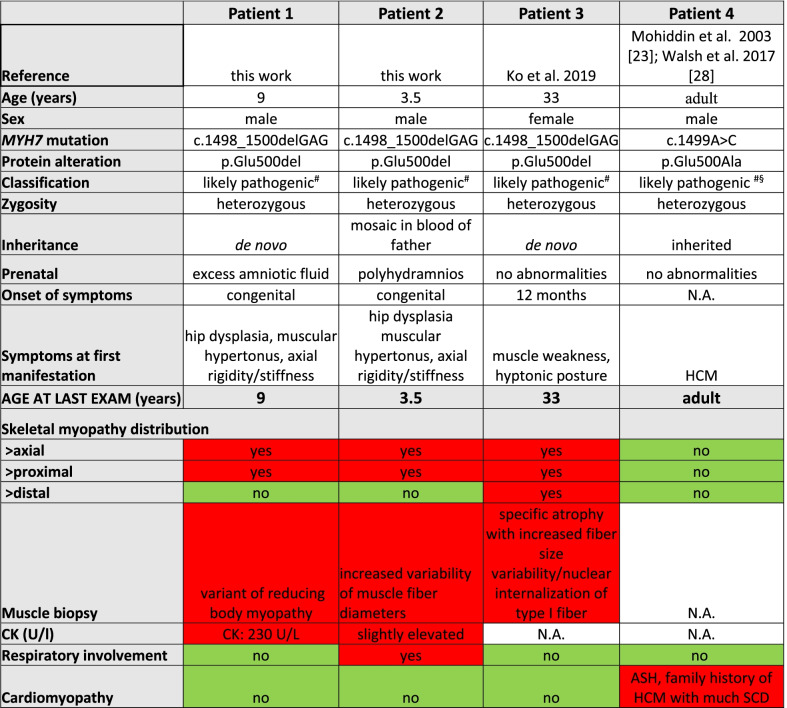
Comparison of all four patients in the literature with a mutation at *MYH7*-residue p.Glu500

Color-code for bottom rows: red = feature was present; green = feature was absent; white = feature was not investigated; HCM = hypertrophic cardiomyopathy; ASH = atrial septal hypertrophy; SCD = sudden cardiac death; N.A. = not available

^#^Classification of the variant according to the recommendations of the ACMG (American College of Medical Genetics); ^#§^ccording to ACMG criteria, the missense variant can be classified as likely pathogenic as it fulfils 2 moderate and ≥ 2 supporting criteria for pathogenicity, i.e. PM1 = well-established functional domain, PM2 = absent from controls, PP2 = missense variant in a gene that has a low rate of benign missense variation, PP3 = pathogenic computational verdict based on twelve pathogenic predictors and no benign predictions. ^§^clinical classification as given in Walsh et al. 2017 on the basis of a single patient is: VUS favors pathogenic [13] (VUS = variant of uncertain significance). An entry in ClinVar (SCV002177799.1) classifies the variant as VUS according to Sherloc [14]

At 9 years of age, MRI of the hip showed symmetrical atrophy of the adductor muscles and of distinct hip muscles (Fig. 1G, H, I, J), i.e. almost complete fatty replacement of adductor longus, adductor brevis, obturator externus, quadratus femoris and only moderate atrophy of adductor magnus.

Creatine kinase (CK) was slightly elevated (230 U/L).

Cardiological investigations at 9 years gave normal results, i.e. normal sinus rhythm and normal repolarization, no signs of hypertrophy, normal contractility.

Neurologic examination at 9 years revealed a rigid spine and hunched shoulders. The patient exhibited hyperlordosis of the cervical spine and kyphosis of the thoracic spine. Active movements in the shoulder-girdle were reduced, abduction of the shoulder was severely limited (~ 20°), passive movements were painful. Pronation and supination in the elbow joints were slightly restricted. Strength of biceps, triceps and the distal muscles was normal. Adduction in the hip joint was severely limited against resistance as was anteflexion against resistance. He performs well at school.

### Patient 2

Patient 2 (Table 1) was born at 37 weeks by elective Caesarean section. The pregnancy was complicated by polyhydramnios. After birth, muscular stiffness and problems in respiratory adaptation were noted. At 3 months a tracheostoma was placed for further respiratory care. During his first months of life, no changes of axially pronounced muscle stiffness were noted. Pharmacologic treatments included baclofen and benzodiazepines without change of muscle tonus.

On examination, he showed visual and mimic reactions to external stimuli.

Inguinal hernia, luxation of both hips and gastro-esophageal reflux were treated surgically.

MRI, EEG, nerve conduction and EMG were normal. Cardiac and metabolic assessment revealed no abnormalities. CK was slightly elevated initially; other laboratory tests were within normal ranges for age.

At 3 years old, the patient continues to show distinct muscle stiffness with rigidity of the spine and contractures of the shoulders, elbows, hips, knees and ankles. He is able to sit, to move himself in a supine position and to ride in a wheelchair. The patient reacts with laughing, mimics, shows emotions and says “mama”. He is able to speak phonetically with a tracheostoma canula and is fed by a jejunal tube. During the night he has non-invasive respiratory support.

### Exome sequencing, sequence analyses and segregation analyses

**Fig. 2:**
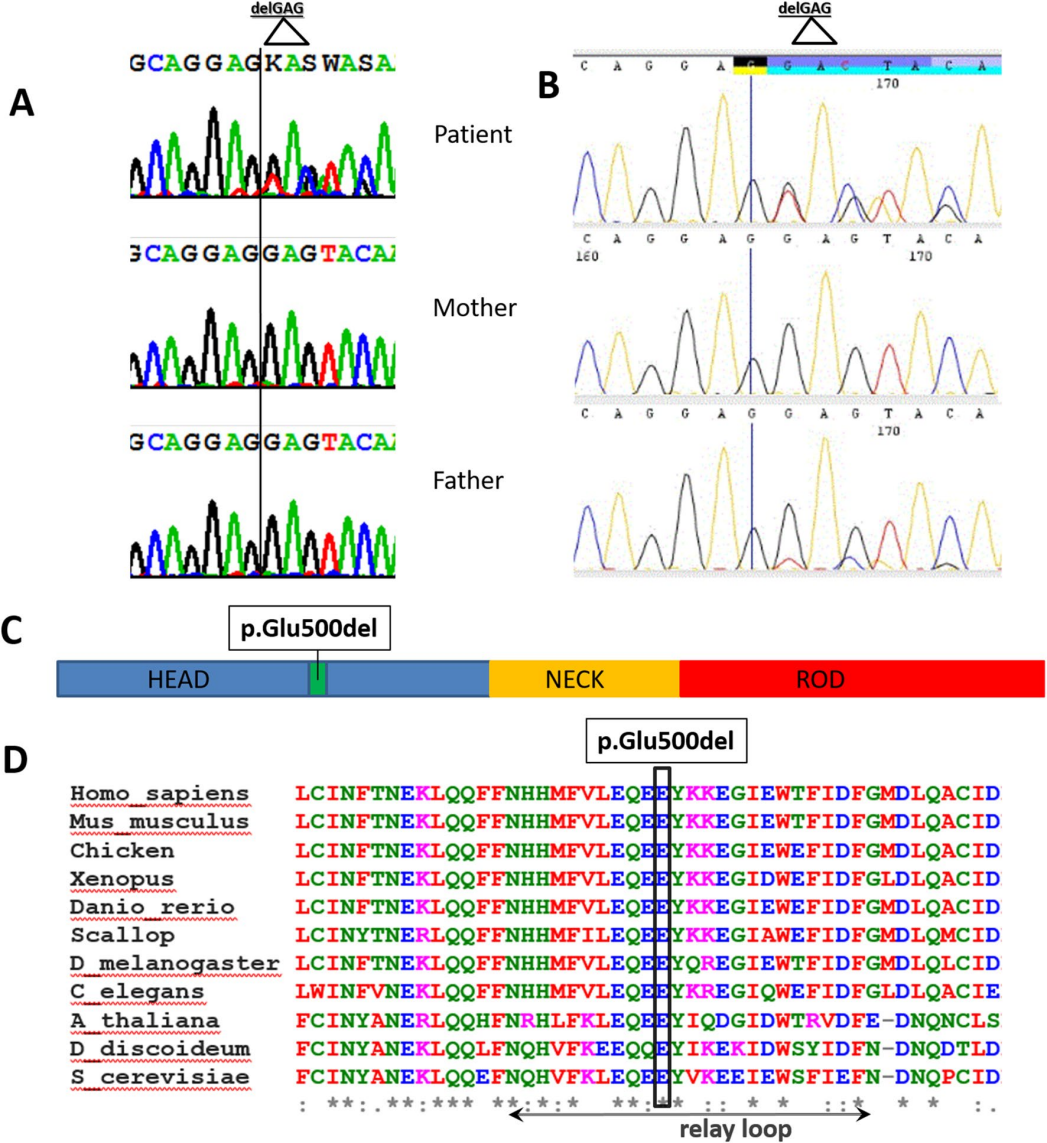
*MYH7* mutation p.Glu500del. (A + B) Electropherograms from Sanger sequencing of DNA from blood of **A** patient 1 (top row) and his parents show the 3-bp in-frame deletion in exon 15: *MYH7* (NM_000257.3) c.1498_1500delGAG p.(Glu500del) heterozygous, de novo. **B** Patient 2 (top row) and his parents: the 3-bp in-frame deletion in exon 15 is present in patient 2 (top row) and also in a fraction of approx. 38% in DNA from blood of the father, as estimated from the area under the curve of the three bases after the frameshift in the electropherogram (bottom row). **C** Graphic representation of the 1935-amino-acid human beta myosin heavy chain polypeptide and location of p.Glu500del. Domains adapted from Colgrave 2014 and derived from the reference-sequence NM_000248.2: head domain (aa 1–847, blue) including the actin-binding domain (aa 655-677 and 757–771), relay-loop (aa 490–513, green), neck-domain (aa 848–1216, orange), rod-domain (aa 1217–1935, red). **D** MSA of *Myh7* orthologs: *Homo sapiens* (NP_000248.2), *Mus musculus* (NP_542766.1); chicken, *Gallus gallus* (NM_001001302); *Xenopus laevis* (NM_001091682); *Danio rerio* (NP_001070932.2); scallop, *Argopecten irradians* (X55714.1); *Caenorhabditis elegans* (NP_724006.1), *Drosophila melanogaster* (NP_724006.1), *Arabidopsis thaliana* (NP_188630.1), *Saccharomyces cerevisiae* (NP_014971.1), *Dictyostelium discoideum* (XP_645195.1), *Saccharomyces cerevisiae* (NP_014971.1). The deleted amino acid position p.Glu500del is indicated by a box. The conserved relay loop (aa 490–513) is indicated by a double-headed arrow

Exome analysis from blood revealed in both unrelated patients a heterozygous in-frame 3-bp deletion in exon 15 of *MYH7* (NM_000257.4): c.[1498_1500delGAG];[ =]; (p.[Glu500del];[ =]). Segregation analysis of the variant showed that the deletion was absent in blood of the parents of patient 1 (Fig. 2A). In the case of patient 2, segregation analysis confirmed mosaicism of the deletion in blood of the father. Based on calculations from sequence traces (electropherograms), we estimate the level of mosaicism in peripheral blood of the father to be ~ 38% (Fig. 2B). The father was not obviously clinically affected.

The deletion was present in dbSNP (rs1555338254) but absent from the gnomAD database (date November 16th 2021)[15] and absent in > 25,000 exomes of the Munich Exome Data Base. Multiple Sequence Alignment (MSA) shows that the mutation deletes an evolutionarily highly conserved (including in invertebrates, fungi and plants) glutamic acid residue (phyloP100way = 7.63) in the relay loop of the globular head domain (Fig. 2C + D, Fig. 4).

### Muscle pathology

**Fig. 3:**
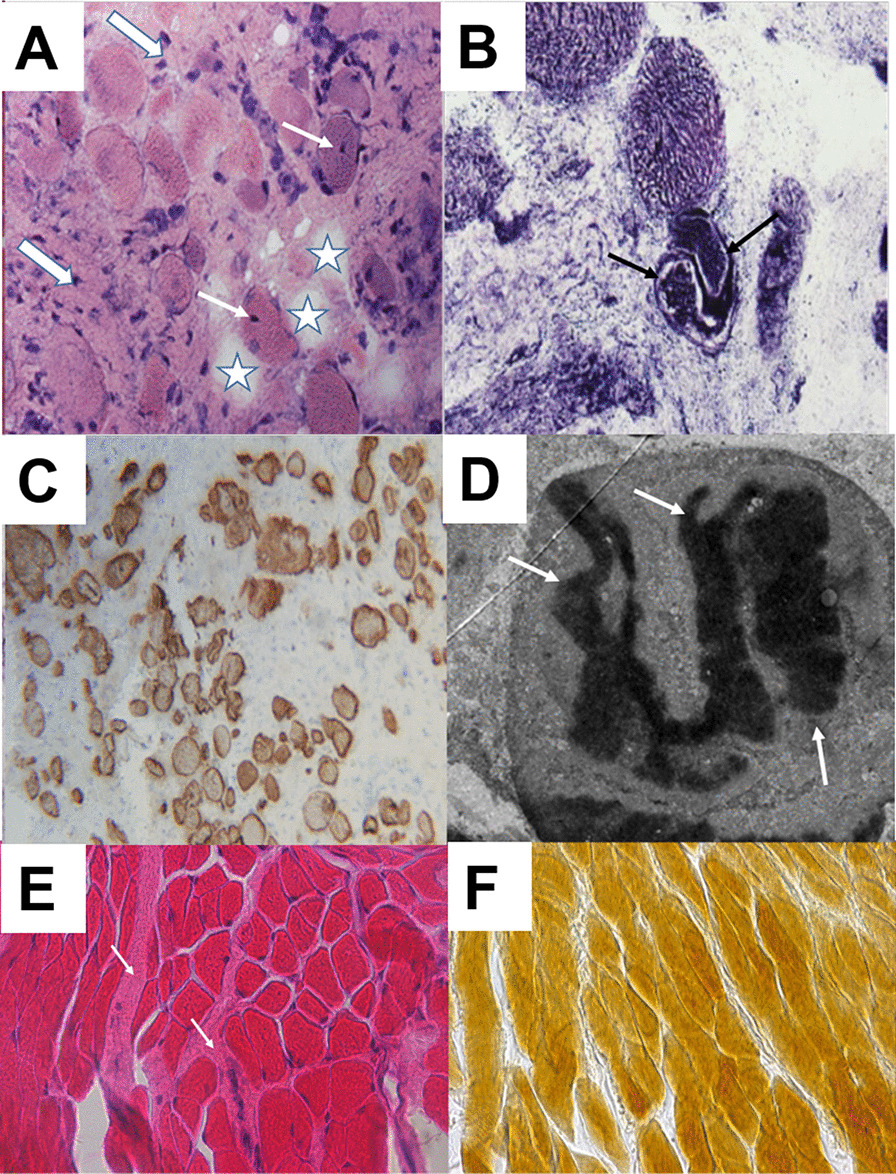
**A**–**D** Patient 1: Stained muscle biopsy from adductor muscle at the age of 18 months. **A** Note increased variability of muscle fiber diameters, endomysial fibrosis (open arrows), endomysial fat cells (asterisks) and muscle fibers with internal myonuclei (arrows). **H** and **E**. **B** Staining for nicotinamide adenine dinucleotide tetrazolium reductase (NADH-TR): note a-reactive, irregularly shaped sarcoplasmatic structures (arrows) displaying a reducing body. **C** Immunohistochemistry using an antibody against type-I myosin displays type-I fiber predominance. **D** Electron microscopy: electrodense, granulofilamentous sarcoplasmic structures resembling a “reducing body” (arrows). **(E + F) Patient 2: Stained muscle biopsy** (**E**) **H** and **E**: Increased variability of muscle fiber diameters and proliferation of endomysial connective tissue (arrows). (**F**) Cytochrome-c-oxidase: uniformly stained muscle fibers compatible with type-I fiber predominance

Muscle biopsies were done in both patients and the observed pathological changes are summarized in Table 1 and Fig. 3. The biopsy of adductor muscle performed in patient 1 at the 1.5 years showed an advanced state of muscular degeneration, resembling the disease known as reducing body myopathy (Table 1, Fig. 3A–D). Muscle biopsy in patient 2 showed increased variability of muscle fiber diameters, proliferation of endomysial connective tissue, and type-I fiber predominance (Fig. 3E + F).

## Discussion

### A causative role for p.Glu500del

Our data indicate that a phenotype of rigidity, stiffness and muscular hypertonia in newborns can be caused by mutations in *MYH7*, similar to the effect of rare, distinct mutations in *TPM3* and *ACTA1*, whose alterations usually cause muscle weakness [16, 17] (Additional file 1: Table S1). The uncommon phenotype and the unexpected mutation site in both of our patients may raise concerns as to the causative role of p.Glu500del in the observed muscular disorder. However, the following three criteria, each suggesting moderate pathogenicity (PM), are met and their combination argues strongly in favor of a pathogenic role of p.Glu500del, according to recommendations of the American College of Medical Genetics and Genomics (ACMG) [18], as follows: PM1—variant is located in a well-established functional domain, PM2—variant is absent from controls, and PM6—variant arose de novo, paternity assumed but not confirmed. Thus, by meeting three criteria of moderate pathogenicity, the variant p.Glu500del can be classified as likely pathogenic (class 4) [18].

### Evolution of the phenotype: from muscular hypertonia to atrophy of axial/proximal muscle groups. And beyond?

The disease in patient 1 was first noted as axial rigidity and stiffness with muscular hypertonia and evolved into a phenotype of rigid spine, atrophy of the spinal paravertebral and neck muscles with involvement of proximal shoulder and hip muscles, resulting in reduced mobility in the shoulder-girdle and the hip. There was no sign of foot drop or other weakness of distal muscles, no signs of cardiomyopathy, and despite marked atrophy of the adductors and hip muscles (Fig. 1), the 9-year-old boy goes on mountain walks and plays soccer.

Weakness limited to axial/proximal muscle groups is exceptionally rare in *MYH7-*related skeletal myopathies. For example, Dabaj et al. recruited patients with *MYH7-*related severe axial involvement and found that all eight patients also had significant weakness of the lower or upper limbs [5].

Cranial, axial and proximal myopathy alone, without any distal involvement, was reported in a 58-year-old woman carrying a missense-mutation in the globular head region (p.Arg249Gln); however, this woman also had HCM [8].

Considering the early presentation of muscular hypertonia, rigidity and stiffness in patient 1, it cannot be excluded that p.Glu500del causes a gain of contractile force. Since hypercontractility and hyperdynamic contraction is a first step in the pathogenesis of HCM [1], it also cannot be excluded that the variant predisposes to HCM, potentially becoming symptomatic later in life. In this context, it is notable that the age of onset of *MYH7-*associated cardiomyopathy can be very early in life, including at birth [6, 7] or prenatally [19], whereas in adult cardiology, patients most commonly present with symptoms during their 3rd and 6th decades [20].

### Possible dual molecular consequences of p.Glu500del

More than 1165 different *MYH7-*mutations are documented in the Human Gene Mutation Database (HGMD [21]) (date June 27th 2021), with missense mutations being the predominant pathogenic mutational type, accounting for 92.4% (1036) of all *MYH7* mutations listed in HGMD. Only 40 small deletions in *MYH7* are documented in HGMD, 11 of which are located in the head domain (8 in-frame deletions and 3 frameshift mutations).

All deletions located in the head domain have been associated with cardiomyopathy, except for one recent case report by Ko et al. [22] that describes a 33-year-old female with early-onset muscular weakness and severe kyphoscoliosis and who possesses the same *MYH7*-mutation (p.Glu500del) as we found in our patients. Thus, our work brings to three the number of unrelated individuals who have this particular deletion (Table 1).

The single amino acid deletion is predicted to be located in the relay loop [23] of the actin-binding head domain, which specifies the converter and lever arm positions. The 3-bp in-frame deletion detected in our patients is situated in a short run of an incomplete purine-rich triplet-repeat (GAG-CAG-GAG-GAG encoding Glu-Gln-Glu-Glu). The mutation was de novo in two families (in our patient 1 and in the patient of Ko et al. [22]) and mosaic in the blood of the father of our patient 2. Considering the recurrence in three unrelated families and the repetitive nature of the respective DNA stretch, it is possible that the affected repeat constitutes a mutational hotspot, similar to other short repeats for which a loss or a gain of a repeat unit is a relatively frequent mutational event [24].

**Fig. 4:**
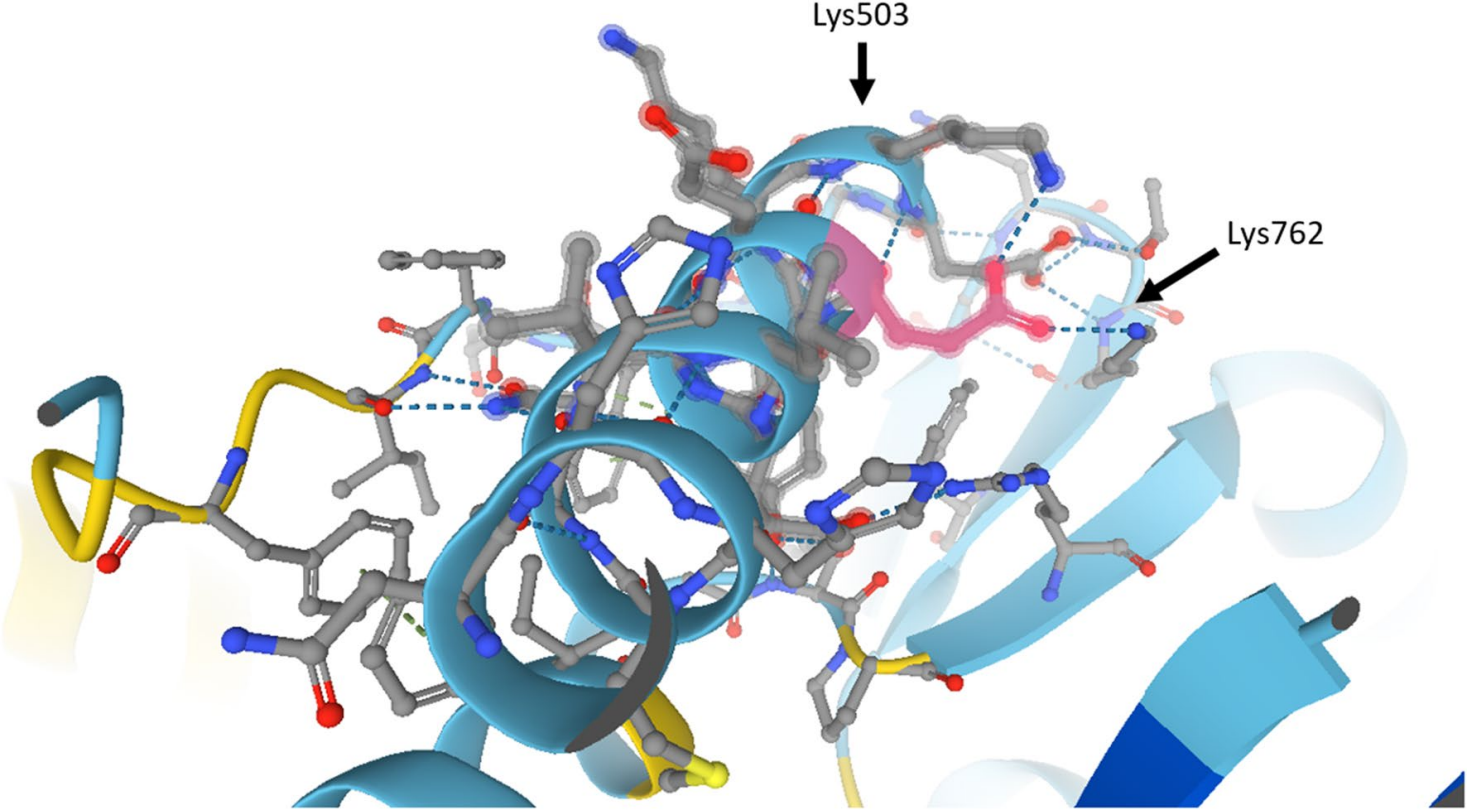
Intramolecular interactions involving p.Glu500, as predicted by AlphaFold. Close-up on the alpha-helix of the relay loop. p.Glu500 (colored in pink) is situated close to one end of the helix and is expected to engage in electrostatic interactions (dashed blue lines) with Lysine503 and Lysine762. Deletion of p.Glu500 may abolish these interactions and dislocate the positively charged amino group of Lysine503

As glutamic acid residues are negatively charged, deletion of one of the evolutionarily conserved consecutive glutamic acid residues (Fig. 2B) would likely alter the charge of the relay loop (Figs. 2D, 4).

The crystal structure of the myosin head (4DB1, Cardiac human myosin S1dC, beta isoform complexed with Mn-AMPPNP, Klenchin et al., 2012, unpublished data, retrieved from Protein Data Bank in Europe) reveals that p.Glu500 is located close to the tip of the alpha helix of the relay loop (data not shown). Based on a prediction by the program AlphaFold [11], p.Glu500 engages in salt bridges with Lysine503 and Lysine762 (Fig. 4). The finding of the heterozygous missense variant NM_000257.4(*MYH7*):c.1499A > C (p.Glu500Ala) in a single patient with HCM, but not skeletal myopathy (Table 1) [13, 25], may indicate that loss of a negative charge at that particular site disrupts these salt bridges, thereby inducing structural changes that ultimately lead to a net increase in myocyte contractility, a feature found in HCM.

Moreover, other missense mutations in the immediate vicinity of p.Glu500 also cause (presumably) isolated cardiomyopathy [25, 26], suggesting that missense variants at this particular site of the protein generally do not cause skeletal myopathy and that additional factors determine whether or not variations of the relay loop ultimately lead to skeletal or cardiac myopathy or both.

In this context it is worth noting that, within an alpha-helical structure, the loss of a single amino acid could dislocate charged amino acids at sites further downstream, such as Lysine503 in the case of Glu500del (Fig. 4). It is therefore tempting to speculate that, independently of the loss of negative charge, Glu500del causes additional structural alterations. These changes may also affect the position of the converter domain [27], thereby perturbing force transmission via the lever arm toward the myosin rod. Finally, variants that affect the coiled-coil rod of the myosin heavy chain dimer have frequently been found to cause skeletal myopathy, either in isolation or, less frequently, accompanied by cardiomyopathy.

## Conclusion

Our findings broaden the phenotypic spectrum of *MYH7*-related myopathies and have implications for early diagnosis in affected families. The hypothesis of a change in sarcomere contractility due to p.Glu500del warrants investigation, which would likely involve the use of patient-derived induced pluripotent stem cells. Over the longer term, the results of these experiments could inform prognosis and therapeutic decisions. At present, it remains elusive whether carriers of p.Glu500del might benefit from the therapeutic application of small molecules that can either activate or repress the slow/cardiac myosin heavy chain [27, 28].

## WEB-Resources

http://genome.ucsc.edu/

http://www.ncbi.nlm.nih.gov/cdd

https://www.ebi.ac.uk/Tools/msa/clustalo/

https://www.ncbi.nlm.nih.gov/clinvar/variation/

## Supplementary Information

**Additional file 1**: **Table 1**. OMIM entries for the three sarcolemmal genes MYH7, ACTA1, TPM3. First column: gene-symbol. Second column: OMIM gene number. Third column: phenotypic entities associated with mutation of the respective gene. Fourth column: OMIM number associated with the respective phenotype. Fifth column: trait mode of inheritance (AD=autosomal dominant; AR=autosomal recessive)

## Data Availability

All data generated or analyzed during this study are included in this published article and its supplementary information files.
