# Supplementary material for: A recurrent single-amino acid deletion (p.Glu500del) in the head domain of ß-cardiac myosin in two unrelated boys presenting with polyhydramnios, congenital axial stiffness and skeletal myopathy
Source: Orphanet J Rare Dis. 2022 Jul 19;17:279. doi: 10.1186/s13023-022-02421-7 (PMC9295345; doi:10.1186/s13023-022-02421-7)
Supplement: Supplementary file 1 — Additional file 1: Table 1. OMIM entries for the three sarcolemmal genes MYH7, ACTA1, TPM3. First column: gene-symbol. Second column: OMIM gene number. Third column: phenotypic entities associated with mutation of the respective gene. Fourth column: OMIM number associated with the respective phenotype. Fifth column: trait mode of inheritance (AD=autosomal dominant; AR=autosomal recessive) [file 13023_2022_2421_MOESM1_ESM.docx]

**Supplementary Material**

| **Gene** | **OMIM** | **Phenotype** | **OMIM** | **Trait** |
| --- | --- | --- | --- | --- |
| *MYH7* | 160760 | Cardiomyopathy, dilated, 1S | [613426](https://omim.org/entry/613426) | AD |
|  |  | Cardiomyopathy, hypertrophic, 1 | [192600](https://omim.org/entry/192600) | AD |
|  |  | Laing distal myopathy | [160500](https://omim.org/entry/160500) | AD |
|  |  | Left ventricular noncompaction 5 | [613426](https://omim.org/entry/613426) | AD |
|  |  | Myopathy, myosin storage, autosomal dominant | [608358](https://omim.org/entry/608358) | AD |
|  |  | Myopathy, myosin storage, autosomal recessive | [255160](https://omim.org/entry/255160) | AR |
|  |  | Scapuloperoneal syndrome, myopathic type | [181430](https://omim.org/entry/181430) | AD |
| *ACTA1* | 102610 | Myopathy, scapulohumeroperoneal | [616852](https://www.omim.org/entry/616852) | AD |
|  |  | Myopathy, actin, congenital, with cores | [161800](https://www.omim.org/entry/161800) | AD, AR |
|  |  | Myopathy, actin, congenital, with excess of thin myofilaments | [161800](https://www.omim.org/entry/161800) | AD, AR |
|  |  | Myopathy, congenital, with fiber-type disproportion 1 | [255310](https://www.omim.org/entry/255310) | AD, AR |
|  |  | Nemaline myopathy 3, autosomal dominant or recessive | [161800](https://www.omim.org/entry/161800) | AD, AR |
| *TPM3* | 191030 | CAP myopathy 1 | [609284](https://www.omim.org/entry/609284) | AD, AR |
|  |  | Myopathy, congenital, with fiber-type disproportion | [255310](https://www.omim.org/entry/255310) | AD, AR |
|  |  | Nemaline myopathy 1, autosomal dominant or recessive | [609284](https://www.omim.org/entry/609284) | AD, AR |

**Supplemental Table 1: OMIM entries for the three sarcolemmal genes *MYH7, ACTA1, TPM3*.** First column: gene-symbol. Second column: OMIM gene number. Third column: phenotypic entities associated with mutation of the respective gene. Fourth column: OMIM number associated with the respective phenotype. Fifth column: trait mode of inheritance (AD=autosomal dominant; AR=autosomal recessive)
